# Dynamics of Hydrogen Peroxide Accumulation During Tip Growth of Infection Thread in Nodules and Cell Differentiation in Pea (*Pisum sativum* L.) Symbiotic Nodules

**DOI:** 10.3390/plants13202923

**Published:** 2024-10-18

**Authors:** Anna V. Tsyganova, Artemii P. Gorshkov, Maxim G. Vorobiev, Igor A. Tikhonovich, Nicholas J. Brewin, Viktor E. Tsyganov

**Affiliations:** 1 Laboratory of Molecular and Cell Biology, All-Russia Research Institute for Agricultural Microbiology, 196608 Saint Petersburg, Russia; a.gorshkov@arriam.ru (A.P.G.); i.tikhonovich@arriam.ru (I.A.T.); vetsyganov@arriam.ru (V.E.T.); 2 Research Park, Saint Petersburg State University, 199034 Saint Petersburg, Russia; vorobiev.maxim@spbu.ru; 3 John Innes Centre, Norwich NR4 7UH, UK; nick.brewin@gmail.com

**Keywords:** hydrogen peroxide, polar growth, infection thread, root nodule, *Rhizobium*–legume symbiosis, *Pisum sativum* L.

## Abstract

Hydrogen peroxide (H_2_O_2_) in plants is produced in relatively large amounts and plays a universal role in plant defense and physiological responses, including the regulation of growth and development. In the *Rhizobium*–legume symbiosis, hydrogen peroxide plays an important signaling role throughout the development of this interaction. In the functioning nodule, H_2_O_2_ has been shown to be involved in bacterial differentiation into the symbiotic form and in nodule senescence. In this study, the pattern of H_2_O_2_ accumulation in pea (*Pisum sativum* L.) wild-type and mutant nodules blocked at different stages of the infection process was analyzed using a cytochemical reaction with cerium chloride. The observed dynamics of H_2_O_2_ deposition in the infection thread walls indicated that the distribution of H_2_O_2_ was apparently related to the stiffness of the infection thread wall. The dynamics of H_2_O_2_ accumulation was traced, and its patterns in different nodule zones were determined in order to investigate the relationship of H_2_O_2_ localization and distribution with the stages of symbiotic nodule development in *P. sativum*. The patterns of H_2_O_2_ localization in different zones of the indeterminate nodule have been partially confirmed by comparative analysis on mutant genotypes.

## 1. Introduction

Reactive oxygen species (ROS), such as superoxide anion (O_2_^•−^), and hydrogen peroxide (H_2_O_2_) are redox-signaling molecules produced by plants in response to environmental cues [1].

One of the most studied ROS is hydrogen peroxide [2,3,4,5,6,7,8]. Numerous studies have shown that H_2_O_2_ plays an important role in plant adaptation to abiotic and biotic stresses [9,10]. It is involved in many resistance mechanisms such as the strengthening of the plant cell wall and the production of phytoalexins. Indeed, numerous studies have shown the H_2_O_2_-dependent cross-linking of cell wall extensins and the participation of hydrogen peroxide in the cleavage of polysaccharides, leading to cell wall modification and different growth responses [8]. H_2_O_2_ has also been shown to act as a signaling molecule, participating in the regulation of a wide range of plant life processes, such as senescence [4,11], photorespiration and photosynthesis [12], stomata movement [13], cell cycle [14], and growth and development [2,15,16,17]. An excessive accumulation of hydrogen peroxide can lead to oxidative stress in the plant, causing cell death [6]. Plant growth and development largely depend on the activation of an effective H_2_O_2_-scavenging mechanism [18,19,20]. Enzymes such as superoxide dismutase, catalase, peroxidase, ascorbate peroxidase, and glutathione reductase [21] are jointly involved in H_2_O_2_ detoxification, as well as non-enzymatic antioxidants such as tocopherols, ascorbic acid, and glutathione [3,12,22,23,24,25,26,27]. Maintaining H_2_O_2_ concentration at an appropriate level can promote plant development and enhance tolerance to environmental stresses. In addition, H_2_O_2_ alters the expression of various genes [28,29]. Numerous studies have shown that H_2_O_2_ is not only itself a key signaling molecule [5,18,30] but also activates many other important signaling molecules in plants (Ca^2+^, salicylic acid, abscisic acid, jasmonic acid, ethylene, and NO) [4,9,30]. These signaling molecules function together and play a complex role in signal transduction pathways during plant growth, development, and stability.

ROS are absolutely essential for the successful development of the *Rhizobium*–legume symbiosis from the earliest stages of its establishment [1,31,32]. Respiratory burst oxidases (Rbohs) have been proposed as a plant source of H_2_O_2_ in nodules [33,34,35,36]. It has been shown that RbohA and RbohB may play a key role in successful rhizobia colonization, apparently as a result of the stimulation of ROS production [34,35]. They are involved in the proper growth and shape of infection threads but do not play a major role in intercellular infection [37]. Class III peroxidases (Prx-IIIs), also called rhizobial-induced peroxidases (Rip1–10), are also considered as potential sources of enzymatic ROS [38,39,40]. Diamine oxidase is another plant source of hydrogen peroxide in nodules that can lead to the cross-linking of tyrosine residues of arabinogalactan protein-extensin molecules, resulting in the hardening of the matrix of infection threads and intercellular space [41]. Bacteria carefully control H_2_O_2_ levels through the activity of catalases [42,43], glutathione [44,45,46], and glutaredoxins [47].

In determinate nodules, H_2_O_2_ was detected both in the intercellular spaces of cortical cells and in infected cells [48,49]. In mature indeterminate nodules, hydrogen peroxide was detected around bacteria in infection threads and in the walls of some infection threads, as well as in the intercellular space and cell walls of cortical cells [43,50,51]. Thus, ROS are essential for the optimal establishment of symbiosis, and they are produced as a specific response to infection associated with the developmental program of both types of nodules [3]. Nevertheless, it should be noted that data on hydrogen peroxide localization in nodules remain fragmentary and contradictory. So far, no studies have been conducted to investigate the localization of hydrogen peroxide in all zones of indeterminate nodule.

In the present study, we analyzed the dynamics of hydrogen peroxide accumulation in wild-type *P. sativum* nodules from four different well-characterized genotypes and corresponding symbiotic mutants blocked at different stages of nodule development.

## 2. Results

### 2.1. Dynamics of Distribution of Hydrogen Peroxide in Infection Threads

**Figure 1:**
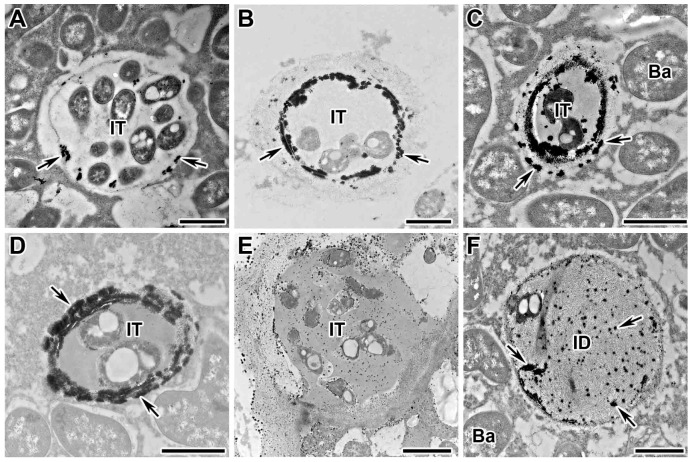
Cytochemical detection of hydrogen peroxide (H_2_O_2_) in sections of wild-type nodules of *Pisum sativum*. Cytochemical reaction with cerium chloride in 2-week-old wild-type line SGE nodules. (**A**) Initial H_2_O_2_ deposits on the inner surface of the infection thread wall. (**B**) Solid deposits of H_2_O_2_ on the inner surface of the infection thread wall. (**C**) Appearance of H_2_O_2_ deposits on the outer surface of the infection thread wall. (**D**) Complete H_2_O_2_ impregnation of the infection thread wall. (**E**) Filling of the infection thread wall and matrix with small crystals of cerium perhydroxide and appearance of precipitates around rhizobia. (**F**) Appearance of cerium perhydroxide precipitates in the matrix of infection threads and droplets. IT, infection thread; ID, infection droplet; Ba, bacteroid; arrows indicate cerium perhydroxide deposits. Bar = 1 µm.

In this study, the dynamics of H_2_O_2_ accumulation in infection threads and droplets was analyzed using cytochemical reaction with cerium chloride in sections of wild-type pea nodules from four different genotypes (laboratory lines SGE and Sprint-2; cultivars ‘Finale’ and ‘Sparkle’). It was shown that H_2_O_2_ started to be deposited on the inner surface of the infection thread wall as individual islets (Figure 1A and Figure S1A,F,K) and then as a continuous layer on the inner surface (Figure 1B and Figure S1B,G,L). Further deposition occurs as islands on the outer surface of the wall (Figure 1C and Figure S1C,H,M) and throughout the thickness (Figure 1D and Figure S1D,I,N). The final action in the modification of the infection thread is the deposition of small cerium perhydroxide crystals in the wall, in the matrix, and around the bacteria within the infection thread (Figure 1E). The matrix of the infection droplet is also filled with numerous cerium chloride precipitates (Figure 1F and Figure S1E,J,O).

### 2.2. Dynamics of Distribution of Hydrogen Peroxide in Cells of Wild-Type Nodules

**Figure 2:**
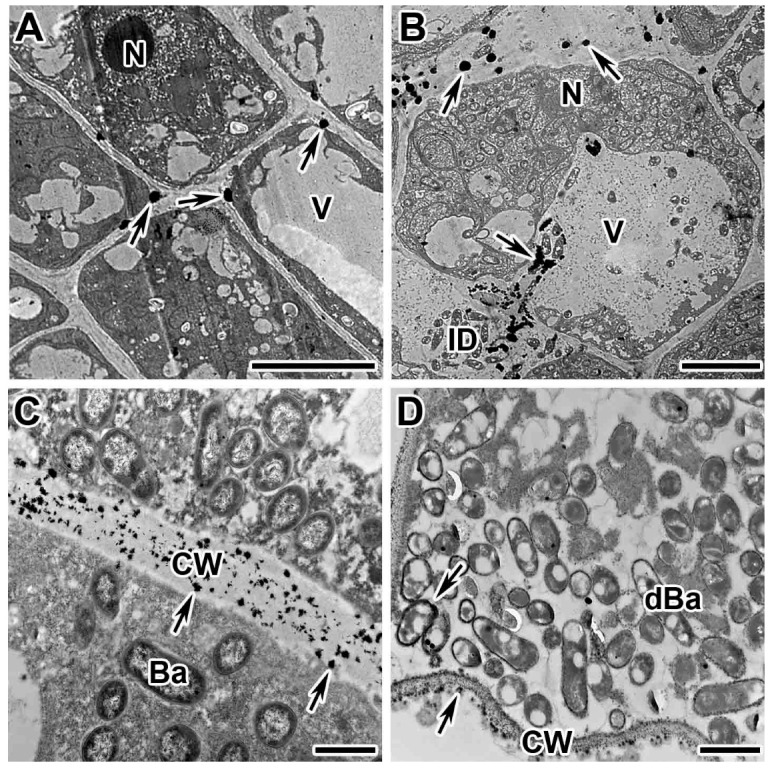
Cytochemical detection of hydrogen peroxide (H_2_O_2_) in wild-type nodules of *Pisum sativum*. Cytochemical reaction with cerium chloride in 2-week-old wild-type nodules of cv. ‘Finale’. (**A**) Meristematic cells. (**B**) Infected cells from the infection zone. (**C**) Infected cells from the nitrogen fixation zone. (**D**) Individual degenerating infected cells from the late nitrogen fixation zone. N, nucleus; V, vacuole; CW, cell wall; ID, infection droplet; Ba, bacteroid; dBa, degenerated bacteroid; arrows indicate cerium perhydroxide deposits. Bar (**A**,**B**) = 5 μm, (**C**,**D**) = 1 μm.

**Figure 3:**
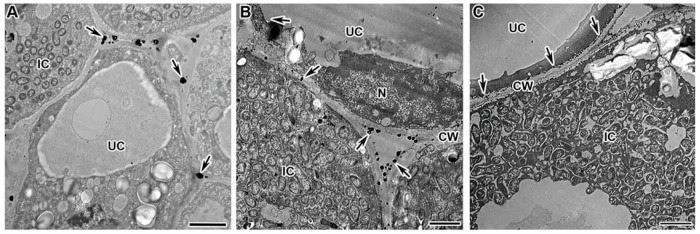
Cytochemical detection of hydrogen peroxide (H_2_O_2_) in wild-type nodules of *Pisum sativum*. Cytochemical reaction with cerium chloride in 2-week-old wild-type nodules: line SGE (**C**) and cv. ‘Finale’ (**A**,**B**). (**A**) Uninfected cell from the infection zone. (**B**) Uninfected cell from the early nitrogen fixation zone. (**C**) Uninfected cell from the late nitrogen fixation zone. IC, infected cell; UC, uninfected cell; N, nucleus; CW, cell wall; arrows indicate cerium perhydroxide deposits. Bar (**A**,**C**) = 1 µm, (**B**) = 2 µm.

In addition to localization in infection structures, the dynamics of hydrogen peroxide distribution was studied in different cell types in various nodule zones in wild types. As a result, it was revealed that in all zones, except for the senescence zone, H_2_O_2_ accumulation was associated with plant cell walls. It was demonstrated that in the meristem (Figure 2A and Figure S2A,E) and the infection zone (Figure 2B and Figure S2B,F), hydrogen peroxide accumulation occurred in individual, or a few, cerium perhydroxide drops located between the plasma membrane and the cell wall. In the early nitrogen fixation zone, the drops became smaller and more numerous (Figure S2C,G). In the late nitrogen fixation zone, the walls of infected cells became impregnated with small crystals of cerium perhydroxide (Figure 2C and Figure S2D,H). In some degenerated infected cells, the deposition of cerium perhydroxide precipitates becomes more intense (Figure 2D), and even more crystals of cerium perhydroxide appeared in the cytoplasm around the symbiosomes (Figure 2D). In the cell walls of the uninfected cells, the pattern of hydrogen peroxide localization in different nodule zones corresponds to that in the infected cells (Figure 3).

### 2.3. Dynamics of Distribution of Hydrogen Peroxide in Infected Cells of Mutant Nodules

The patterns of H_2_O_2_ localization in different zones of the wild-type indeterminate nodule were partially confirmed by comparative analysis on *P. sativum* symbiotic mutants. Nevertheless, a detailed analysis of mutants blocked at different developmental stages revealed features of hydrogen peroxide localization associated with the mutant phenotype.

The mutants in the gene *Sym33* are characterized with “locked” infection threads with thickened suberized walls [52,53] and thickened suberized cell walls [53], as well as the presence of suberized cell wall material in the vacuole [54]. In addition, bacteria are dead inside the infection threads of the nodules of mutant *sym33-2* [55]. These traits indicate strongly expressed defense responses in these mutants.

**Figure 4:**
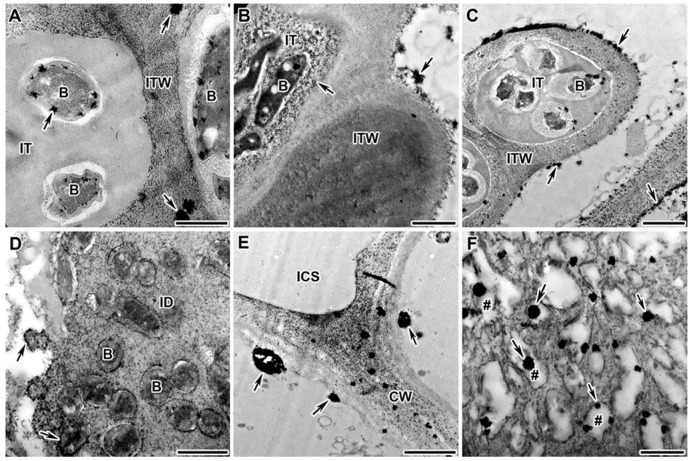
Cytochemical detection of hydrogen peroxide (H_2_O_2_) in nodules of *Pisum sativum* mutants in the *Sym33* gene. Cytochemical reaction with cerium chloride in 2-week-old nodules of mutants SGEFix^–^-2 (*sym33-3*) (**A**,**C**,**D**,**F**), RBT4 (*sym33-3*, *sym42*) (**B**), and RBT3 (*sym33-3*, *sym40-1*) (**E**). (**A**,**B**) Infection threads with different intensity of cerium perhydroxide precipitate accumulation. (**C**) Degraded rhizobia in the infection thread. (**D**) Infection droplet completely filled with cerium perhydroxide precipitates. (**E**) Cell wall of colonized cell. (**F**) Transport vesicles with cerium perhydroxide precipitates. CW, cell wall; ICS, intercellular space; IT, infection thread; ID, infection droplet; ITW, infection thread wall; B, bacterium; #, vesicle; arrows indicate cerium perhydroxide deposits. Bar (**C**) = 2 µm, (**B**,**D**,**E**) = 1 μm, (**A**,**F**) = 500 nm.

When studying the localization and distribution of hydrogen-peroxide-colonized cells in the nodules of mutants in gene *Sym33*, an excessive accumulation of H_2_O_2_ was demonstrated in the form of small cerium perhydroxide crystals in the infection thread walls (Figure 4A,B), around bacteria (Figure 4A,B), in the infection thread matrix (Figure 4C), and in infection droplets (Figure 4D), as well as in cell walls (Figure 4E). In addition, vesicles carrying cell wall material to the plasma membrane were detected with cerium perhydroxide crystals (Figure 4F).

**Figure 5:**
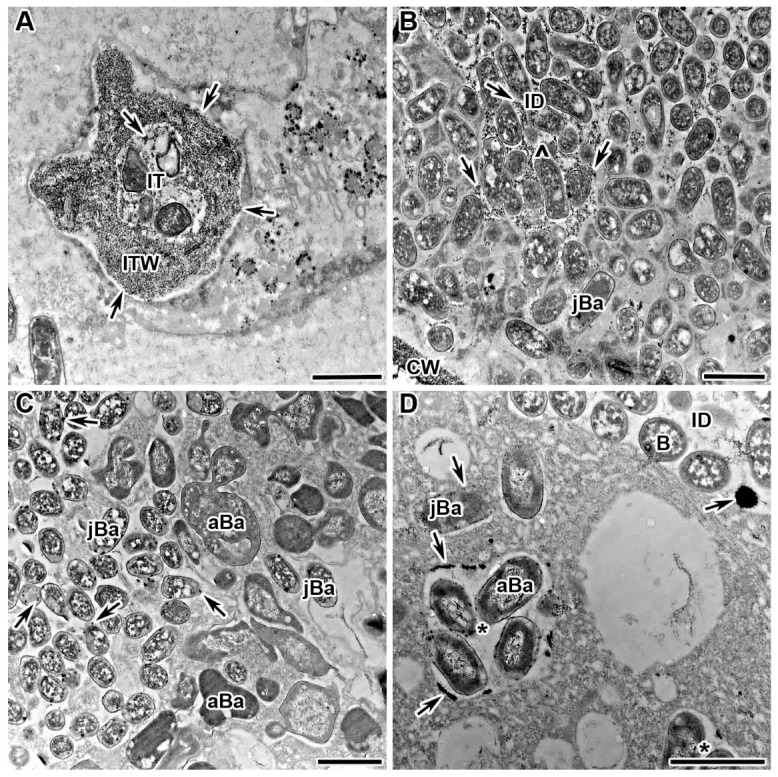
Cytochemical detection of hydrogen peroxide (H_2_O_2_) in nodules of *Pisum sativum* mutant in the *Sym40* gene. Cytochemical reaction with cerium chloride in 2-week-old nodules of the mutant SGEFix^–^-1 (*sym40-1*). (**A**) Infection thread with intense accumulation of cerium perhydroxide precipitates in the wall and matrix. (**B**) Infection droplet completely filled with cerium perhydroxide precipitates. (**C**) Juvenile bacteroids with cerium perhydroxide precipitates and further formation of abnormal bacteroids. (**D**) Formation of multibacteroid symbiosomes surrounded by cerium perhydroxide precipitates. CW, cell wall; IT, infection thread; ID, infection droplet; ITW, infection thread wall; B, bacterium; jBa, juvenile bacteroid; aBa, abnormal bacteroid; ^, infection droplet matrix filled with small cerium perhydroxide precipitates; *, multibacteroid symbiosome; arrows indicate cerium perhydroxide precipitates. Bar = 1 μm.

The mutant SGEFix^–^-1 (*sym40-1*) is characterized with hypertrophied infection droplets and abnormal bacteroid differentiation, as well as the strong defense reactions manifested in the suberization of the nodule endodermis [52,53]. In this study, the nodules of the mutant SGEFix^–^-1 (*sym40-1*) were characterized by an excessive accumulation of H_2_O_2_ in the infection thread walls (Figure 5A), in the matrix of hypertrophied infection droplets (Figure 5B), around some juvenile bacteroids (that form further abnormal bacteroids) (Figure 5C), and in the symbiosome membrane in multibacteroid symbiosomes (Figure 5D).

**Figure 6:**
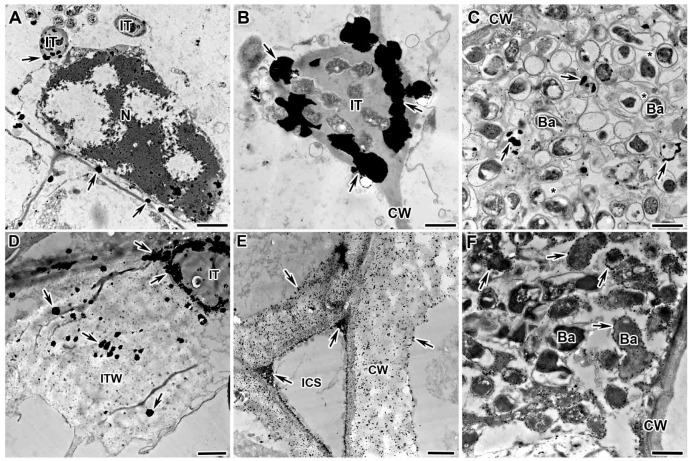
Cytochemical detection of hydrogen peroxide (H_2_O_2_) in nodules of *Pisum sativum* mutants. Cytochemical reaction with cerium chloride in 2-week-old nodules of the mutants Sprint-2Fix^–^ (*sym31*) (**A**–**C**) and RisFixV (*sym42*) (**D**–**F**). (**A**) Small cerium perhydroxide precipitates in nuclear heterochromatin. (**B**) Infection thread abundantly filled with cerium perhydroxide precipitates. (**C**) Small cerium perhydroxide precipitates around bacteroids in individual and multibacteroid symbiosomes. (**D**) Infection thread with a thickened callose-impregnated wall with large cerium perhydroxide precipitates. (**E**) Cell wall with callose deposits filled with small cerium perhydroxide precipitates. (**F**) Small cerium perhydroxide precipitates around degenerated bacteroids in senescent infected cell. N, nucleus; CW, cell wall; IT, infection thread; ITW, infection thread wall; ICS, intercellular space; Ba, bacteroid; *, multibacteroid symbiosome; arrows indicate cerium perhydroxide precipitates. Bar = 1 μm.

The mutant Sprint-2Fix^–^ (*sym31*), characterized by undifferentiated bacteroids and clustered into multibacteroid symbiosomes [56], showed an abnormal accumulation of small cerium perhydroxide crystals in the nuclear heterochromatin (Figure 6A), an excessive accumulation of H_2_O_2_ in the infection thread matrix (Figure 6B), and the appearance of small cerium perhydroxide precipitates around bacteroids in individual and multibacteroid symbiosomes (Figure 6C).

The mutant RisFixV (*sym42*) is known for the formation of infection threads with thickened callose-impregnated walls and morphologically differentiated bacteroids that undergo premature senescence [53,57,58]. For the mutant RisFixV (*sym42*), hydrogen peroxide was shown to accumulate around prematurely senescent bacteroids (Figure 6F) in the form of large drops of cerium perhydroxide in the thickness of the callose-impregnated wall of infection threads (Figure 6D) and small crystals in thickened cell walls (Figure 6E).

## 3. Discussion

Reactive oxygen species (ROS), both radicals and non-radical active molecules produced by oxygen oxidation, are associated with numerous adaptive responses and development in both animal and plant cells [59]. ROS are produced both in stress-resistance reactions and during physiological metabolism [6,60]. During evolution, plants have been able to achieve a high degree of control over the accumulation of ROS and, in particular, H_2_O_2_ [61].

In the *Rhizobium*–legume symbiosis, during infection, the production of superoxide anion (O_2_^•−^) and H_2_O_2_ has been observed in infection threads and infected cells [39,50,51]. In this case, hydrogen peroxide accumulation is suggested to promote the hardening of the infection thread matrix as a result of the cross-linking of tyrosine residues of arabinogalactan protein extensin molecules [41]. Thus, the role of hydrogen peroxide in nodule development is now attributed to a signaling function in the early stages of interaction [5] and an increase in the stiffness of the infection thread matrix necessary for its successful growth [3,5].

**Figure 7:**
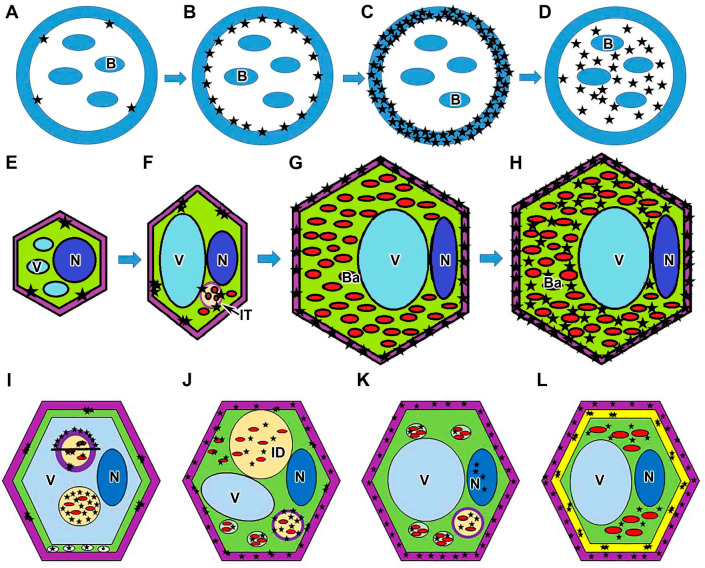
Schematic showing the dynamics of hydrogen peroxide accumulation in symbiotic nodule. (**A**–**D**) Sequential stages of hydrogen peroxide accumulation in the walls and matrix of the infection thread. B, bacterium; infection thread wall is presented in blue; black stars indicate cerium perhydroxide precipitates. (**E**–**H**) Sequential stages of hydrogen peroxide accumulation in the cell walls of meristematic cell (**E**), colonized cell from infection zone (**F**), infected cell from nitrogen fixation zone (**G**), and senescent cell (**H**). (**I**–**L**) Hydrogen peroxide accumulation in nodules of ineffective mutants. (**I**) Distribution of hydrogen peroxide in the nodules of the mutants in gene *Sym33* (two patterns of H_2_O_2_ distribution in infection threads are presented). The thin layer of cytoplasm around the nucleus, infection thread, and infection droplet is not indicated for simplicity (**J**) Distribution of hydrogen peroxide in the nodules of mutant *sym40-1*. (**K**) Distribution of hydrogen peroxide in the nodules of mutant *sym31*. (**L**) Distribution of hydrogen peroxide in the nodules of mutant *sym42*. N, nucleus (dark blue); V, vacuole (light blue); IT, infection thread (light yellow with violet wall); ID, infection droplet (light yellow); Ba, bacteroid (red; symbiosome with a single bacteroid is presented without a membrane, and multibacteroid symbiosome is presented with symbiosome membrane); cell wall is presented in violet; callose cell wall is presented in yellow; peroxisomes are presented in white; cytoplasm is presented in green; black stars indicate cerium perhydroxide precipitates. Objects are not scaled.

In this study, hydrogen peroxide localization was analyzed in nodules of four different pea genotypes. It was found that H_2_O_2_ deposition in the infection threads and infection droplets is characterized by specific dynamic characteristics of all genotypes studied (Figure 7A–D). The observed sequential stages of hydrogen peroxide accumulation help to explain the differences in the previously described patterns of H_2_O_2_ localization in infection threads. For example, in alfalfa (*Medicago sativa*) nodules, hydrogen peroxide was detected between the walls of infection threads and the matrix [43] as well as in the infection thread matrix and infection thread wall [50]. In infection threads in nodules of both alfalfa and pea, H_2_O_2_ was localized around bacteria, in the walls of infection threads, and in “patches” in the matrix of infection threads [51].

The dynamics of hydrogen peroxide deposition in the walls of different types of nodule cells was also analyzed (Figure 7E–H). Earlier, the localization of hydrogen peroxide in cell walls was shown for infected cells in the infection zone but has not been described in the cell walls of meristematic and infected cells in the nitrogen fixation zone in indeterminate nodules [50,51].

At the same time, H_2_O_2_ was not detected in symbiosomes in active nitrogen-fixing cells (Figure 2B,C and Figure S2B–D,G,H). Previously, hydrogen peroxide localization using cerium chloride also failed to detect H_2_O_2_ in symbiosomes in active infected cells. However, histochemical localization using diaminobenzidine revealed hydrogen peroxide in infected cells of *Lotus japonicus* nodules [49]. Moreover, H_2_O_2_ production was detected in *Medicago truncatula* nodules in cells of the inner cortex and infection zone using HyPer, the fluorescent probe for H_2_O_2_ [29].

In this study, in single senescent cells, H_2_O_2_ accumulation around degrading bacteroids was observed (Figure 2D). Previously, in alfalfa, pea, and soybean nodules, a lot of cerium perhydroxide precipitates were also observed around the peribacteroid and bacteroid membranes in senescing infected cells, confirming the involvement of H_2_O_2_ in the senescence process [48,50,51].

Along with the localization of hydrogen peroxide in wild-type nodule cells, its localization in nodule cells of pea symbiotic mutants blocked at different stages of nodule development was studied in this work (Figure 7I–L).

The localization and distribution of hydrogen peroxide was studied in nodules of pea mutants for the *Sym33* gene characterized by “locked” infection threads without bacterial release. It was shown that the pattern of H_2_O_2_ accumulation in mutants for the *Sym33* gene (Figure 7I), into the cytoplasm of plant cells, to some extent corresponds to the pattern of localization in the meristem and the early infection zone in pea wild-type nodules. In addition, this mutant is also characterized by pronounced defense reactions like thickened suberized cell walls and infection thread walls [53], as well as the presence of suberized cell wall material in the vacuole [54], accompanied by excessive hydrogen peroxide accumulation (Figure 7I) and the appearance of numerous peroxisomes (Figure 7I). Indeed, the macromolecular assembly of polyphenolic domains during suberization occurs via a H_2_O_2_-dependent peroxidase-mediated free-radical binding process [62].

In the mutant SGEFix^–^-1 (*sym40-1*), which is characterized by the hypertrophy of infection droplets, an excessive accumulation of cerium perhydroxide precipitates in infection threads and infection droplets was observed (Figure 7J) compared with wild-type nodules. This may be attributed to the development of strong oxidative stress as a result of incompatible interactions due to the mutant phenotype, as observed in plant–pathogen interactions [59,63]. In addition, the deposition of cerium perhydroxide precipitates on the bacterial membranes of juvenile bacteroids recently released into host cytoplasm was also observed in the mutant SGEFix^–^-1 (*sym40-1*) (Figure 7J). Rhizobia (free-living forms) are known to be more sensitive to H_2_O_2_ than other bacterial species [64,65]; however, during differentiation into bacteroids, they can survive utilizing the host antioxidant system to cope with H_2_O_2_. It seems that the bacteroids in SGEFix^–^-1 (*sym40-1*) are unable to overcome this stress, and the differentiation process goes incorrectly, as indicated by the presence of abnormal bacteroids in the cytoplasm of infected nodule cells and their early senescence [66].

The mutant Sprint-2Fix^–^ (*sym31*) is characterized by undifferentiated bacteroids [56]. In this mutant, small crystals of cerium perhydroxide in the cell walls of infected cells were observed resembling H_2_O_2_ accumulation in cell walls in the nitrogen fixation zone in wild-type nodules. In addition, the mutant Sprint-2Fix^–^ (*sym31*) developed oxidative stress as manifested by the appearance of hydrogen peroxide in the nucleus (Figure 7K) and an excessive accumulation of cerium perhydroxide precipitates in the infection thread matrix (Figure 7K) and around undifferentiated bacteroids in multibacteroid symbiosomes (Figure 7K).

In the mutant RisFixV (*sym42*) with a premature senescence of symbiotic structures and pronounced defense reactions in the form of callose deposition in cell walls and infection thread walls [53], different patterns of the deposition of cerium perhydroxide precipitates were observed. In large infected cells in nodules of this mutant, in which bacteroids have not yet degenerated, the pattern of H_2_O_2_ accumulation was similar to that in infected cells in wild-type nodules. Degenerating infected cells in nodules of this mutant were completely filled with cerium perhydroxide precipitates (Figure 7L). The cell walls and callose-impregnated infection thread walls also showed different patterns of H_2_O_2_ localization (Figure 7L); hydrogen peroxide accumulated in the thickness of callose deposits in the form of clusters.

## 4. Materials and Methods

### 4.1. Plant Material and Bacterial Strain

Pea (*Pisum sativum* L.) ineffective (Fix^–^) mutants blocked at different stages of nodule development and corresponding wild types were used (Table 1).

In all experiments, *P. sativum* plants were inoculated with *Rhizobium johnstonii* strain 3841 [74] (former *Rhizobium leguminosarum* bv. *viciae* 3841 strain [75].

### 4.2. Plant Growth Conditions

Seeds were sterilized with concentrated sulfuric acid for 30 min and washed ten times with sterile water. The seeds were planted in pots containing 200 mL of vermiculite and 100 mL nutrient solution without nitrogen [76], and then each seed was inoculated with 1 mL of an aqueous suspension of bacteria (10^7^–10^8^ cells). Plants were grown in a growth chamber MLR-352H (Sanyo Electric Co., Ltd., Moriguchi, Japan) under controlled conditions: day/night, 16/8 h; temperature, 21 °C; relative humidity 75%; photosynthetic photon flux density of ~280 μmol photons m^−2^ s^−1^. For histochemical analysis, three independent experiments were performed. Nodules of *P. sativum* were harvested on day 14 after inoculation (DAI). For each variant, ten nodules from different plants were analyzed.

### 4.3. Histochemical Localization of H_2_O_2_

To detect hydrogen peroxide (H_2_O_2_), a cytochemical reaction with cerium chloride was carried out to form electron-dense deposits of cerium perhydroxide [77]. For this purpose, the nodules were immediately immersed after harvesting in a 10 mM solution of cerium chloride (CeCl_3_) in 50 mM MOPS (3-(N-Morpholino)propanesulfonic acid) (Sigma-Aldrich, St. Louis, MO, USA) solution (pH 7.0) for 1 h in vacuum before fixation in 2.5% glutaraldehyde (Sigma-Aldrich) in 0.1 M cacodylate buffer (pH 7.2) (Sigma-Aldrich). Nodules treated and untreated (negative control) with cerium chloride were additionally fixed for 1 h in a 1% aqueous solution of osmium tetraoxide (OsO_4_) (Electron Microscopy Sciences, Hatfield, PA, USA) in 0.1 M cacodylate buffer and then subjected to routine sample preparation for electron microscopy and embedded in epoxy resin Epon812 (Honeywell Fluka, Waltham, MA, USA) at 60 °C for 48 h. Ultrathin sections (90–100 nm) were contrasted with 2% aqueous uranyl acetate solution (Electron Microscopy Sciences) for 20 min and further contrasted with lead citrate solution [78] for 5 min. For transmission electron microscopy, ultrathin sections (90–100 nm thick) were cut with a Leica EM UC7 ultramicrotome (Leica Microsystems, Vienna, Austria) and counterstained as described previously [66]. Nodule tissues were examined using a JEM-1200 EM transmission electron microscope (JEOL Ltd., Tokyo, Japan) at 80 kV. Electron micrographs were captured with a Veleta CCD camera (Olympus, Münster, Germany).

## 5. Conclusions

In this work, the dynamics of hydrogen peroxide accumulation and patterns of its localization in different zones of pea nodules were studied. The results obtained correlate well with the previously proposed role of hydrogen peroxide in the growth of infection threads through its association with an increase in the stiffness of the infection thread wall. The role of hydrogen peroxide in the maturation of the cell wall of infected cells during their differentiation has also been proposed. The revealed intensive accumulation of hydrogen peroxide in nodules of pea symbiotic mutants reflects the activation of defense reactions and oxidative stress during the development of ineffective symbiosis.

## Figures and Tables

**Table 1:** Plant material used in the study.

Genotype	Nodule Phenotype	References
SGE	Wild type	[52,67]
SGEFix^–^-1 (*sym40-1*) ^1^	Hypertrophied infection droplets and infection threads, abnormal bacteroids, early nodule senescence	[52]
SGEFix^–^-2 (*sym33-3*) ^2^	“Locked” infection threads, absence of bacterial release into the host cell cytoplasm of most infected cells ^3^	[52]
RBT3 (*sym33-3*, *sym40-1*)	“Locked” infection threads, absence of bacterial release	[68]
RBT4 (*sym33-3*, *sym42*)	“Locked” infection threads, absence of bacterial release	[69]
Sprint-2	Wild type	[70]
Sprint-2Fix^–^ (*sym31*)	Undifferentiated bacteroids	[56]
‘Sparkle’	Wild type	[71]
‘Finale’	Wild type	[57,58,72]
RisFixV (*sym42*)	Early nodule senescence, thickening of the infection thread wall	[53,57,58,72,73]

^1^ The *Sym40* gene is orthologous to the *M. truncatula EFD* gene [73]. ^2^ The *Sym33* gene is orthologous to the *M. truncatula IPD3* gene [73]. ^3^ The mutant line *sym33-3* has a leaky phenotype, and bacterial release occurs in some cells or nodules [52].

## Data Availability

Data are contained within the article or Supplementary Materials.

## Supplementary Materials

The following supporting information can be downloaded at https://www.mdpi.com/article/10.3390/plants13202923/s1. Figure S1: Cytochemical detection of hydrogen peroxide (H_2_O_2_) in sections of wild-type nodules of *Pisum sativum* (infection threads); Figure S2: Cytochemical detection of hydrogen peroxide (H_2_O_2_) in wild-type nodules of *Pisum sativum* (nodule cells).
