# Supplementary material for: Dynamics of Hydrogen Peroxide Accumulation During Tip Growth of Infection Thread in Nodules and Cell Differentiation in Pea (Pisum sativum L.) Symbiotic Nodules
Source: Plants (Basel). 2024 Oct 18;13(20):2923. doi: 10.3390/plants13202923 (PMC11510766; doi:10.3390/plants13202923)
Supplement: Supplementary file 1 [file plants-13-02923-s001.zip › plants-3221674-supplementary.pdf]

Electronic Supplementary Material

Dynamics of Hydrogen Peroxide Accumulation During Tip Growth of Infection Thread in Nodules and Cell Differentiation in Pea (*Pisum sativum* L.) Symbiotic Nodules

Anna V. Tsyganova[1,*], Artemii P. Gorshkov[1], Elena V. Seliverstova[1,2], Maxim G. Vorobiev[3], Igor A. Tikhonovich[1,3], Nicholas J. Brewin[4], and Viktor E. Tsyganov[1]

1 All-Russia Research Institute for Agricultural Microbiology, Laboratory of Molecular and Cell Biology, Saint Petersburg, Russia; avtsyganova@arriam.ru (A.V.T.); elena306@yandex.ru (E.V.S.); vetsyganov@arriam.ru (V.E.T.)

2 Sechenov Institute of Evolutionary Physiology and Biochemistry of the Russian Academy of Sciences, Saint Petersburg, 194223, Russia; elena306@yandex.ru (E.V.S)

3 Saint Petersburg State University, 199034, Saint Petersburg, Russia; vorobiev.maxim@spbu.ru (M.G.V.)

4 Formerly Emeritus Fellow, John Innes Centre, Norwich NR4 7UH, UK; nick.brewin@gmail.com (N.J.B.)

*Author for correspondence:

Anna V. Tsyganova

Tel: +7 812 4705100

E-mail: avtsyganova@arriam.ru

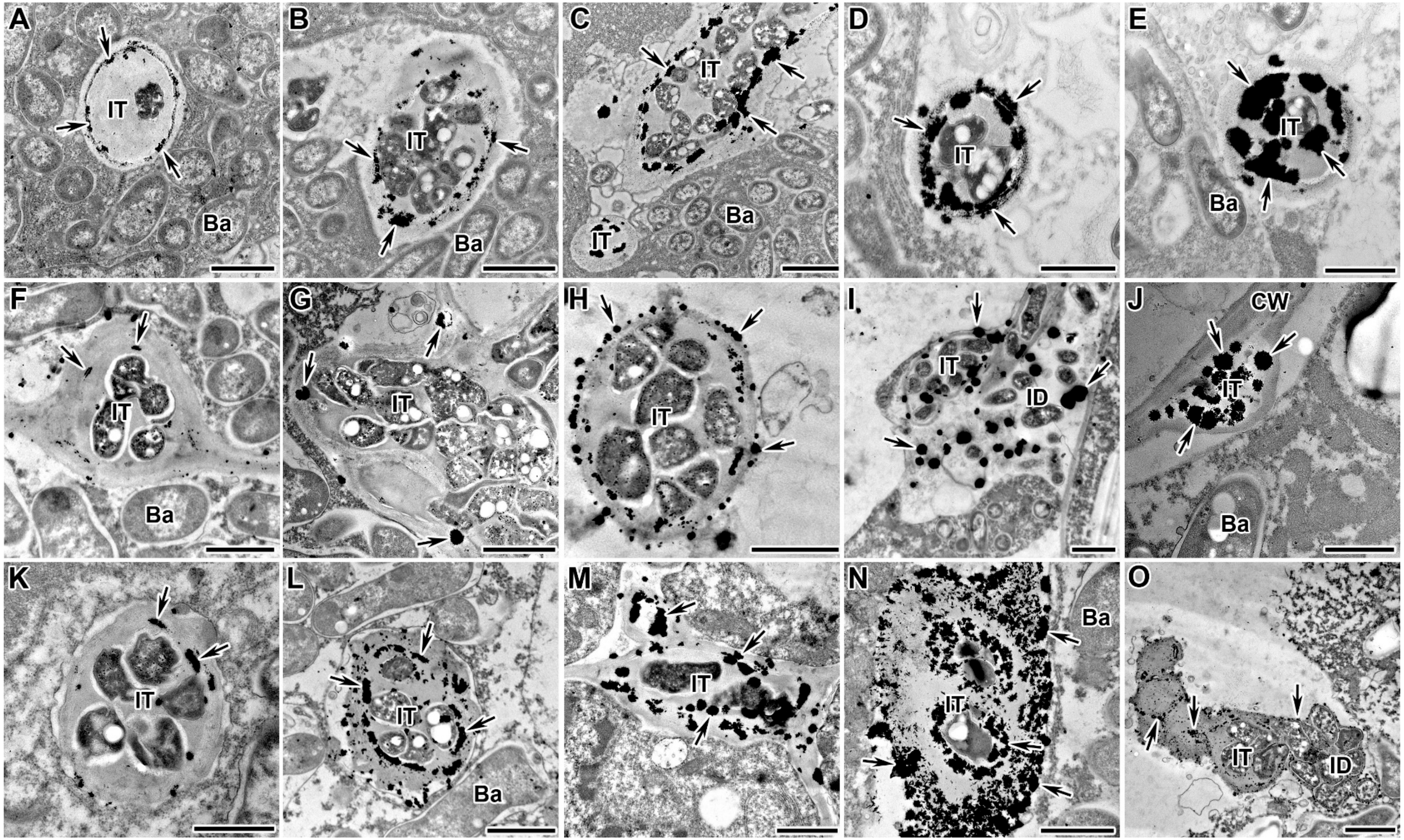

Figure S1. Cytochemical detection of hydrogen peroxide (H_2O_2) in sections of wild-type nodules of *Pisum sativum*. Cytochemical reaction with cerium chloride in 2-week-old wild-type nodules: Finale (A-E), Sprint-2 (F-J), Sparkle (K-O). (A, F, K) Initial H_2O_2 deposits on the inner surface of the infection thread wall. (B, G, L) Solid deposits of H_2O_2 on the inner surface of the infection thread wall. (C, H, M) Appearance of H_2O_2 deposits on the outer surface of the infection thread wall. (D, I, N) Complete H_2O_2 impregnation of the infection thread wall. (E, J, O) Appearance of cerium perhydroxide precipitates in the matrix of infection threads and droplets. IT, infection thread; ID, infection droplet; Ba, bacteroid; CW, cell wall; arrows indicate cerium perhydroxide deposits. Bar (A-I, K-O) = 1 μm, (J) = 500 nm.

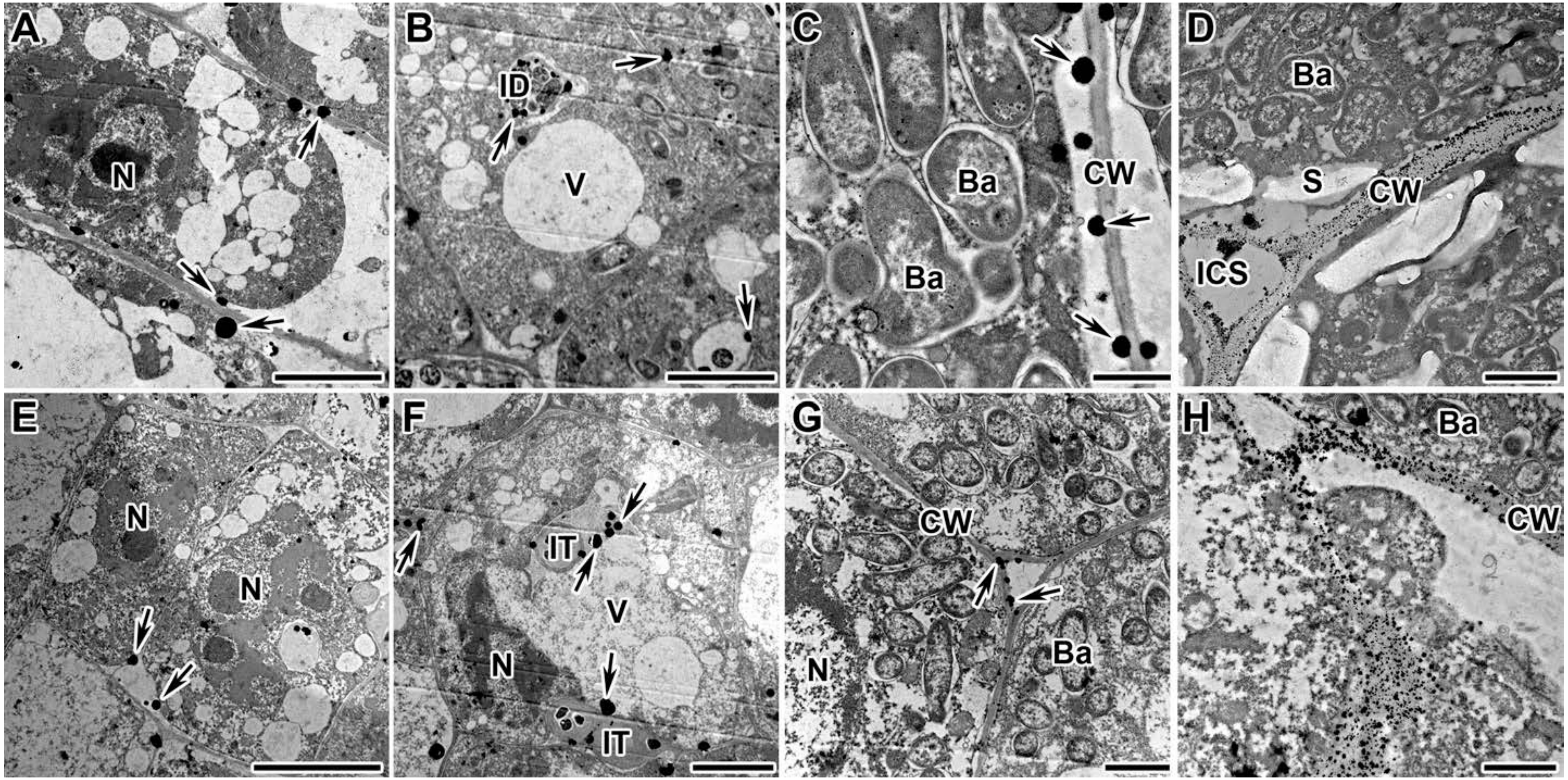

Figure S2. Cytochemical detection of hydrogen peroxide (H_2O_2) in wild-type nodules of *Pisum sativum*. Cytochemical reaction with cerium chloride in 2-week-old wild-type nodules: SGE (A-D), Sprint-2 (E-H). (A, E) Meristematic cells. (B, F) Infected cells from the infection zone. (C, G) Infected cells from the early nitrogen fixation zone. (D, H) Infected cells from the late nitrogen fixation zone. N, nucleus; V, vacuole; CW, cell wall; ICS, intercellular space; IT, infection thread; ITW, infection thread wall; ID, infection droplet; Ba, bacteroid; S, starch; arrows indicate cerium perhydroxide deposits. Bar (A, B, F, G) = 5 μm, (D, E, H-J) = 1 μm, (C) = 500 nm.
